# Supplementary material for: Structural Characterization of Full-Length Human Dehydrodolichyl Diphosphate Synthase Using an Integrative Computational and Experimental Approach
Source: Biomolecules. 2019 Oct 28;9(11):660. doi: 10.3390/biom9110660 (PMC6921004; doi:10.3390/biom9110660)
Supplement: Supplementary file 1 [file biomolecules-09-00660-s001.pdf]

biomolecules

MDPI

Supplementary data for

Structural characterization of full-length human dehydrodolichyl diphosphate synthase using an integrative computational and experimental approach

Michal Lisnyansky Bar-El[1], Su Youn Lee[2], Ah Young Ki[2], Noa Kapelushnik[3], Anat Loewenstein[4], Ka Young Chung[2], Dina Schneidman-Duhovny[5,6], Moshe Giladi[1,7,¶,*], Hadas Newman[4,¶], Yoni Haitin[1,¶,*]

[1] Department of Physiology and Pharmacology, Sackler Faculty of Medicine, Tel-Aviv University, Tel-Aviv, Israel
[2] School of Pharmacy, Sungkyunkwan University, Jangan-gu, Suwon, South Korea
[3] Department of Ophthalmology, Sheba Medical Center, Ramat Gan, Israel
[4] Department of Ophthalmology, Tel-Aviv Sourasky Medical Center, Tel Aviv, Israel
[5] Department of Biological Chemistry, Institute of Life Sciences, The Hebrew University of Jerusalem, Jerusalem, Israel
[6] School of Computer Science and Engineering, The Hebrew University of Jerusalem, Jerusalem, Israel
[7] Tel Aviv Sourasky Medical Center, Tel Aviv, Israel
¶ These authors contributed equally to this work.

* Correspondence: moshegil@post.tau.ac.il (MG), yhaitin@tauex.tau.ac.il (YH)

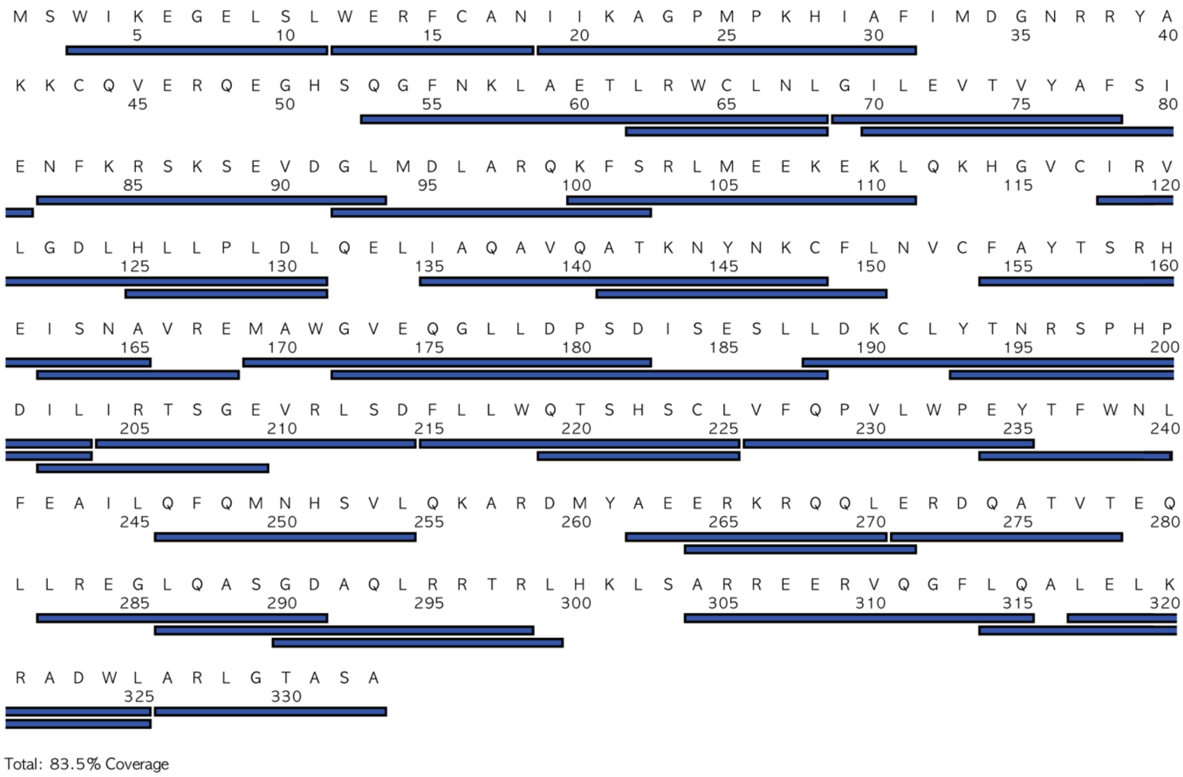

Figure S1. HDX-MS analysis of human DHDDS. Sequence coverage of human DHDDS.

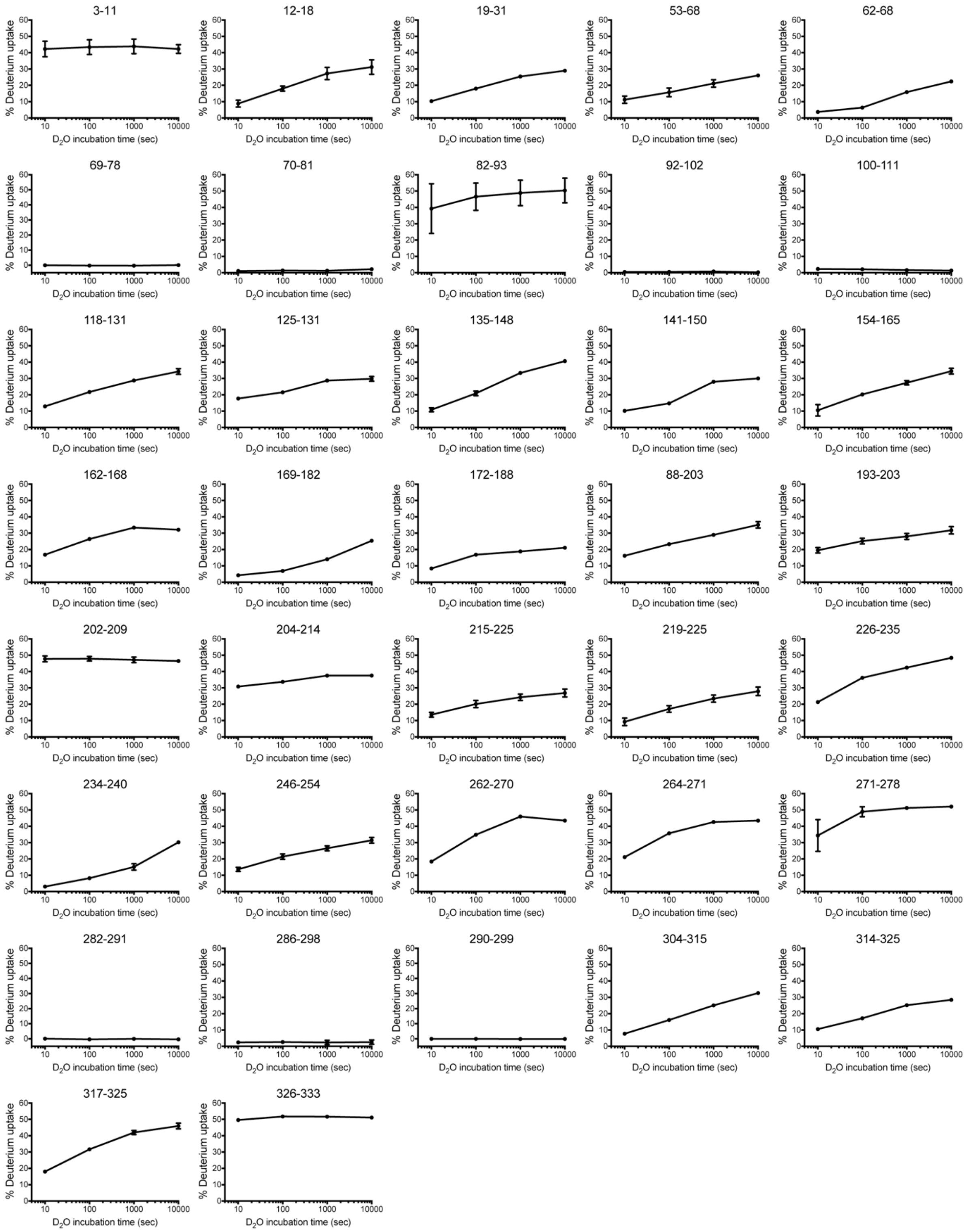

Figure S2. HDX-MS analysis of human DHDDS. Deuterium uptake plots.
